# Safety and efficacy of pharmacotherapy containing INSTIs and chemotherapy drugs in people living with HIV and concomitant colorectal cancer

**DOI:** 10.1186/s12981-022-00470-3

**Published:** 2022-09-23

**Authors:** Jing Yang, Guo Wei, Fuqiang Gui, Yong Zhao, Tingyu Chen, Juan Tan

**Affiliations:** Department of General Surgery and Oncology Surgery, Public Health Clinical Center of Chengdu, Jingju Temple 18#, Chengdu, 610066 China

**Keywords:** Colorectal cancer (CRC), Human immunodeficiency virus (HIV), Antiretroviral therapy (ART), Adverse events (AEs), Integrase inhibitors (INSTIs)

## Abstract

**Background:**

Previous clinical data have shown that raltegravir-based antiretroviral therapy (ART) with fewer drug-drug interactions (DDIs) and adverse events (AEs) is a good regimen in patients with HIV infection who need cancer chemotherapy. There are currently few data on ART regimens that include Integrase inhibitors (INSTIs) other than RAL among this patient subgroup.

**Methods:**

We evaluated the safety and efficacy of different kinds of INSTI-based regimens among patients with HIV and concomitant colorectal cancer (CRC) who received antineoplastic agents.

**Results:**

From January 2020 to November 2021, 66 patients were enrolled. The patients were divided into three groups: 20 patients treated with dolutegravir (DTG)/lamivudine (3TC)/tenofovir (TDF) (group I), 24 patients treated with DTG/albuvirtide (ABT) (group II), and 22 patients treated with bictegravir (BIC)/tenofovir alafenamide (TAF)/emtricitabine (FTC) (group III). The majority of AEs during treatment were of grade 1–2. Treatment‐related AEs of grade 3–4 occurred in 6 patients (9.09%), and no grade 5 AEs occurred. The most common AEs were nausea (100%) and neutrophils (84.85%) attributed to anticancer agents, and there was no significant difference in the incidence of these AEs among the three groups (P > 0.05). Viral load rebound was not observed among pretreated patients during chemotherapy. The viral load of untreated patients who started their ART concomitant with chemotherapy almost decreased to the lower limit of detection 6 months after ART initiation (only one patient in group III had a viral load of 102 copies/ml). At the 6th month, the CD4 count in group I decreased significantly from baseline (P < 0.05). However, the change in CD4 count was not significant in group II (P = 0.457) or group III (P = 0.748).

**Conclusions:**

DTG- or BIC-containing regimens are good options for patients with HIV and concomitant CRC.

## Introduction

The introduction of antiretroviral therapy (ART) prolongs the survival of patients with human immunodeficiency virus (HIV) and turns HIV infection into a chronic condition. An increase in non-AIDS-defining cancers has been documented. Regardless of the type of cancer among patients with HIV, malignancies have become one of the most frequent causes of HIV-related deaths [1–4], among which colorectal cancer (CRC) is the most commonly diagnosed malignancy [5]. The need for CRC treatment and HIV treatment with concurrent antineoplastic agents and antiretrovirals is increasingly common [6]. The key challenge facing oncologists is how to administer chemotherapy effectively and safely to patients who are undergoing antiretroviral therapy. National Comprehensive Cancer Network (NCCN) guidelines state that ART should not be interrupted during antitumor treatment, and continuous ART may lead to better tumor treatment tolerance and better prognosis [7, 8].

However, it has been reported that there may be drug-drug interactions (DDIs) when antiviral drugs are used concurrently with antitumor drugs, which may lead to increased toxicity in the treatment process and even to chemotherapy delays or dose reductions, thereby affecting the prognosis [9]. It has been reported that the incidence of adverse events (AEs) in patients with HIV during chemotherapy is twice that of patients without HIV [10]. However, it is relatively safe to use Integrase inhibitor (INSTI)-based regimens as ART strategies in this situation because INSTIs are better tolerated than protease inhibitors or non-nucleoside reverse transcriptase inhibitors, and they have a low risk of DDIs, low toxicity and low rate of chemotherapy-related side effects [7, 11]. Current clinical studies on ART regimens during chemotherapy are based on raltegravir (RAL), which is the first clinically approved INSTIs for the treatment of HIV [8, 12–14]. In fact, RAL is a first generation INSTI with low drug-related central nervous system symptoms [15]. Few studies have addressed the use of other INSTIs concurrent with chemotherapy. Our research aims to explore the safety and efficacy of ART regimens containing other INSTIs except RAL during chemotherapy among people living with HIV and concomitant CRC.

## Methods

### Patients

We conducted a retrospective cohort study of patients with HIV and stage II-III CRC seen at the Public Health Clinical Center of Chengdu from January 2020 to November 2021. The inclusion criteria were as follows: patients with HIV and concomitant stage II–III CRC confirmed by pathological examination; age between 18 and 85 years old; patients who started or switched from ART to an INSTI-based regimen during chemotherapy; and patients who underwent radical resection of CRC and chemotherapy after operation. The exclusion criteria were as follows: patients who underwent neoadjuvant chemotherapy; patients with complete intestinal obstruction and undergoing emergency surgery; patients complicated with severe liver, kidney, heart, lung, or other organ dysfunction; pregnant or lactating women. The 6 month follow-up period began at the time of chemotherapy initiation.

All patients provided written informed consent before enrollment. The trial protocol and all amendments were approved by the institutional review board of Public Health Clinical Center of Chengdu.

### Safety and efficacy evaluation

CRC clinicopathologic information, treatment information, CRC staging (AJCC 8th standards), ART regimen, chemotherapy regimen, dose reductions and/or delays, AEs, HIV viral load (COBAS AmpliPrep/COBAS TaqMan Analyzer, Roche, Diagnostics, USA, lower limit of detection 20 copies/ml), and CD4 count (BD FacsCalibur analyzer, BD Biosciences, USA) were obtained by medication records and available laboratory data.

The safety evaluation measures by clinical review of all relevant parameters included neutropenia, anemia, thrombocytopenia, liver function damage, kidney function damage, hypersensitivity, diarrhea, nausea, vomiting, fever, and infection. Efficacy evaluation indicators included CD4 count, CD4/CD8 ratio, and HIV viral load. Patients were examined and tested at baseline and every chemotherapy cycle (usually approximately every 3 weeks) by our hospital laboratory. AEs were also reported every chemotherapy cycle and assessed according to the Common Terminology Criteria for Adverse Events, version 4.02.

Treatment was postponed in the case of serious AEs such as peripheral neuropathy ≥ grade 3, myelosuppression ≥ grade 3, etc. If the serious AEs still existed when a dose delay and supportive careoccurred, the antineoplastic drug dose should be reduced. Here, we considered a dose delay to be a chemotherapy postponement of greater than 3 days due to toxicity. Virological failure was defined as HIV viral load > 200 copies/mL ≥ 24 weeks on an ARV regimen initiated or monitored or virological rebound (HIV RNA ≥ 200 copies/mL after virological suppression) [16].

### Statistical methods

The incidence of AEs was calculated, and the severity of AEs was statistically described. The chi square test or Fisher’s test was used for comparison of categorical variables among the 3 groups. A paired t test was used for the change in CD4 count and CD4/CD8 ratio in each group. A p value less than 0.05 was considered statistically significant. All analyses were performed using the Statistical Package of Social Sciences (SPSS) version 23.0 (IBM Corp., Armonk, NY).

## Results

### Demographics and CRC characteristics

**Fig. 1:**
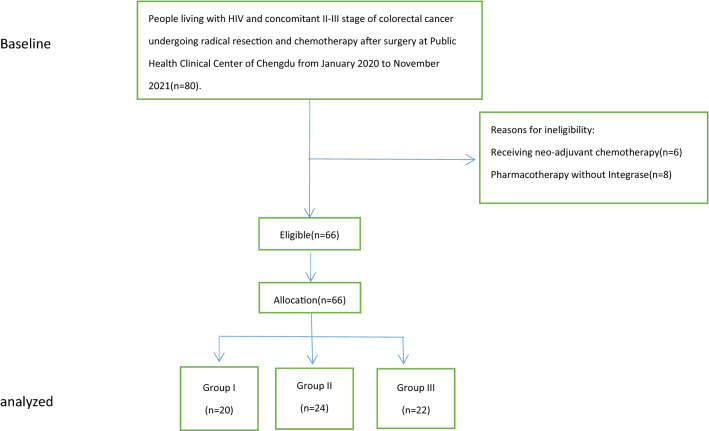
Participant flowchart of patients with HIV and concomitant stage II-III colorectal cancer undergoing radical resection and chemotherapy after surgery at the Public Health Clinical Center of Chengdu from January 2020 to November 2021. We excluded 14 patients who did not meet the inclusion criteria. Ultimately, 66 patients (group I: 20, group II: 24, group III: 22) were eligible and enrolled, and none were lost to follow up

From January 2020 to November 2021, a total of 66 patients with HIV infection and concomitant CRC were identified. The disposition of the patients, including reasons for exclusion, is illustrated in Fig. 1. The average age of all patients was 58.56 ± 10.57 years old. Fifty-eight out of 66 patients were pretreated patients. The remaining patients were all untreated patients. In this study, we defined pretreated patients as patients who were on ART continuously for at least 1 month before chemotherapy initiation. For 58 pretreated patients, ART regimens (including protease inhibitors in 2 and non-nucleoside reverse transcriptase inhibitors in 56) were switched to INSTI-containing regimens before chemotherapy. Patients were divided into 3 groups according to different antiviral regimens during the chemotherapy period: group I (n = 20) dolutegravir (DTG)/lamivudine (3TC)/tenofovir (TDF); group II (n = 24) DTG/albuvirtide (ABT); and group III (n = 22) bictegravir (BIC)/tenofovir alafenamide (TAF)/emtricitabine (FTC). All the pretreated patients maintained virological suppression. In group I, 1 patient was untreated, with the viral load baseline of 7700 copies/ml. In group II, 4 patients were untreated, with viral load baselines of 6170 copies/ml, 13 900 copies/ml, 29 600 copies/ml and 365 000 copies/ml. Three patients in group III were untreated, with viral load baselines of 4710 copies/ml, 8080 copies/ml and 9500 copies/ml. For pretreated patients, those with a viral load of 20–1000 copies/ml at baseline all started ART within 2 months. Fifty-seven patients received CapeOX, and 9 received mFolfox6 as a chemotherapy regimen after surgery. Treatments were administered every three weeks for 8 cycles. Demographic characteristics and basic information are presented in Table 1.

**Table 1:** General characteristics of patients with HIV and concomitant CRC according to antiviral regimens (n = 66)

Demographic characteristics	DTG/3TC/TDF (group I) n = 20	DTG/ABT (group II) n = 24	BIC/TAF/FTC (group III) n = 22
Sex			
Male	13	18	8
Female	7	6	14
Age (yeras)	61.5 (44–74)	60.04 (45–85)	54.27 (33–74)
BMI (kg/m^2^)	23.37 (18.37–28.23)	22.74 (17.97–26.81)	23.73 (18.03–25.30)
TNM stage			
II	14	10	18
III	6	14	4
Differentiation			
Well differentiated	0	3	2
Moderately differentiated	15	14	16
Poorly differentiated	5	7	4
Baseline CD4 count (cells/ul)	368.35 (195–980)	272.71 (83–757)	315.14 (85–980)
CD4/CD8 ratio	0.91 (0.19–2.87)	0.75 (0.14–2.86)	0.60 (0.08–1.68)
Type of cancer			
Rectal	6	11	4
Colon	14	13	18
Comorbidities			
Hypertension	2	1	2
Diabetes mellitus	4	1	3
HBV	1	1	1
HCV	1	2	0
Type of surgery			
Right hemicolectomy	3	9	6
Left hemicolectomy	5	1	4
Sigmoidectomy	6	3	8
Low anterior resection	6	11	4
Baseline HIV viral load (copies/ml)			
≤ 20	18	19	17
20–1000	1	1	2
> 1000	1	4	3
Chemotherapy regimen			
CapeOX	16	19	22
mFolfox6	4	5	0
Untreated patients	1	4	3
Pretreated patients	19	20	19

CRC = Colorectal cancer

DTG/3TC/TDF = dolutegravir/lamivudine/tenofovir

DTG/ABT = dolutegravir/albuvirtide

BIC/TAF/FTC = bictegravir/tenofovir alafenamide/emtricitabine

BMI = Body Mass Index

TNM stage was according to the AJCC 8th classification

According to the medical records and clinical data, we list the common AEs of all patients during treatment (Table 2). Treatment-related AEs of any grade occurred in all 66 patients. The majority of AEs during treatment were of grade 1–2. Treatment‐related AEs of grade 3–4 occurred in 6 patients (9.09%), and grade 5 AEs did not occur. The highest side effects were nausea (100%) and neutrophils (84.85%), which were attributed to anticancer agents. The incidence rate of peripheral neurotoxicity, manifesting paresthesia of fingers mainly, was 53.03% and all were grade 1–2. 60 patients developed nausea in the first chemotherapy cycle. The median time to first onset among patients was 15 weeks (the 5th chemotherapy cycle) for patients with neutrophils and 12 weeks (the 4th chemotherapy cycle) for patients with peripheral neurotoxicity. No significant differences in the AE incidence rate among the groups were detected (P > 0.05). Four patients in group II and group III had a slight increase in transaminase (grade I–II), and there were no cases of rebound of hepatitis B or hepatitis C viral load. Two patients in group II had a slight decrease in left ventricular systolic function (Table 2).

**Table 2:** Types of AEs in patients with HIV and concomitant CRC according to antiviral regimens (n = 66)

AEs	DTG/3TC/TDF (group I) n = 20		DTG/ABT (group II) n = 24		BIC/TAF/FTC (group III) n = 22		P value
	Any grade	grade 3-5	Any grade	Grade 3-5	Any grade	Grade 3-5	
Neutropenia	19	1	21	1	16	1	0.129
Anemia	4	0	3	1	7	0	0.284
Thrombocytopenia	2	0	1	0	1	0	0.672
Peripheral neurotoxicity	13	0	11	0	11	0	0.421
Fever	1	0	1	0	0	0	–
Infection	0	0	0	0	1	0	–
Hypersensitivity	0	0	1	0	0	0	–
Liver function damage	0	0	1	0	3	0	–
Renal Impairment	1	0	1	0	0	0	–
Nausea	20	1	24	0	22	1	–
Vomiting	2	1	2	0	3	0	0.884
Diarrhea	0	0	0	0	1	0	–
Impaired cardiac function	0	0	2	0	0	0	–

AEs = Adverse events

CRC = Colorectal cancer

DTG/3TC/TDF = dolutegravir/lamivudine/tenofovir

DTG/ABT = dolutegravir/albuvirtide

BIC/TAF/FTC = bictegravir/tenofovir alafenamide/emtricitabine

P value meant statistical difference of AEs in any grade among the three groups

In group III, one patient developed tuberculosis in the 6th cycle of chemotherapy, so chemotherapy was interrupted and antituberculosis therapy was initiated. In group II, one patient developed grade 4 neutropenia accompanied by fever in the fourth chemotherapy cycle, resulting in dose delay (5 days) and reductions (Table 3). The patient had resolution of the event after receiving granulocyte colony-stimulating factor and supportive treatment.

**Table 3:** Dose reduction and/or delay in patients with HIV and concomitant CRC according to antiviral regimens (n = 66)

	DTG/3TC/TDF (group I) n = 20	DTG/ABT (group II) n = 24	BIC/TAF/FTC (group III) n = 22
Delayed chemotherapy	0	1	0
Dose reduction of chemotherapy	0	1	0
Interruption of chemotherapy	0	0	1

CRC = colorectal cancer

DTG/3TC/TDF = dolutegravir/lamivudine/tenofovir

DTG/ABT = dolutegravir/albuvirtide

BIC/TAF/FTC = bictegravir/tenofovir alafenamide/emtricitabine

All patients took antiviral drugs regularly during chemotherapy and no patient had virological failure. Rebound viral load did not occur among pretreated patients. The viral load of untreated patients who started their ART concurrent with antineoplastic drugs almost decreased to the lower limit of detection half a year after ART initiation, and only one patient in group III whose baseline was 9500 copies/ml, had a detected viral load level of 102 copies/ml in the 6th month. At the 6th month, the CD4 count in group I decreased significantly from baseline (P < 0.05) and there was no statistical difference in the change of CD4/CD8 ratio (P = 0.139). However, the change in CD4 count was not obvious in group II (P = 0.457) or group III (p = 0.748) (Table 4).

**Table 4:** The change of CD4 count and HIV viral load in patients with HIV and concomitant CRC according to antiviral regimens (n = 66)

		Baseline	M1	M3	M6
DTG/3TC/TDF (group I) n = 20	CD4 (cells/ul)	368.35 (195–980)	319.2 (164–624)	280.6 (146–578)	247.5 (150–456)
	CD4/CD8 ratio	0.91 (0.19–2.87)	0.72 (0.22–2.22)	0.69 (0.3–1.64)	0.69 (0.5–1.01)
	HIV viral load (copies/ml)				
	≤ 20	18	19	20	20
	20–1000	1	1	1	0
	> 1000	1	0	0	0
DTG/ABT (group II) n = 24	CD4 (cells/ul)	272.71 (83–757)	262.29 (108–494)	267.25 (210–513)	301 (167–337)
	CD4/CD8 ratio	0.75 (0.14–2.86)	0.53 (0.11–0.8)	0.52 (0.2–1.4)	0.67 (0.2–1.5)
	HIV viral load (copies/ml)				
	≤ 20	19	22	24	24
	20–1000	1	1	0	0
	> 1000	4	1	0	0
BIC/TAF/FTC (group III) n = 22	CD4 (cells/ul)	315.14 (85–980)	404.27 (111–479)	388.91 (86–577)	327.38 (82–612)
	CD4/CD8 ratio	0.60 (0.08–1.68)	0.98 (0.09–2.86)	0.92 (0.21–1.7)	0.76 (0.2–1.29)
	HIV viral load (copies/ml)				
	≤ 20	17	20	20	21
	20–1000	2	1	1	1
	> 1000	3	1	1	0

*CRC* colorectal cancer

DTG/3TC/TDF = dolutegravir/lamivudine/tenofovir

DTG/ABT = dolutegravir/albuvirtide

BIC/TAF/FTC = bictegravir/tenofovir alafenamide/emtricitabine

M1 = the first month ± 1 week

M3 = the third month ± 1 week

M6 = the sixth month ± 1 week

## Discussion

The dearth of clinical understanding of potential DDIs and chemotherapy tolerability in patients with HIV is largely due to this subgroup typically being excluded from cancer clinical trials. It has been reported that patients with HIV tolerate chemotherapy poorly, and subsequent dose reductions and/or delays affect their cancer treatment outcomes [10]. Another study showed that chemotherapy with 5-fluorouracil (5-FU) and cisplatinum was also feasible with low toxicity in patients with HIV treated with antiretroviral therapies [17]. In this study, most AEs were mild and well tolerated, and the incidence of AEs was similar among the three groups. Consistent with previous studies, neutropenia were one of the common side effects attributed to anticancer agents [18, 19]. Previous studies showed that, in CRC patients without HIV, nausea/vomiting and neutropenia were also common adverse reactions. The incidence were about 40–69% and 41–67.5% respectively [20, 21], which were lower than that of HIV patients (100% and 84.85%) in this study. Peripheral neurotoxicity is a frequent side effect of oxaliplatin treatment, and previous studies showed that the incidence of oxaliplatin-related neurotoxicity was up to 50–80% [22, 23]. In this study, the incidence rate of sensory alteration or paresthesia was 53.03% and did not interfere with function. One patient in group III developed tuberculosis in her chemotherapy period with a CD4 count of 93 cells/µl and an undetected viral load level. Therefore, patients with low CD4 counts are likely to experience opportunistic infection and have a poorer tolerance for therapy. In addition, the CD4 count decreased significantly from baseline only in group I. Therefore, for patients with a low CD4 baseline, it is very important to have a early intervention, supportive care and management of AEs, which could minimize unnecessary treatment discontinuations in general.

The majority of antiviral drugs (protease inhibitors and non-nucleoside reverse transcriptase inhibitors) and chemotherapeutic agents are metabolized via the cytochrome P450 (CYP450) pathway [24]. Therefore, the potential interaction of chemotherapeutic agents with antiviral drugs is great [25]. Nucleoside reverse transcriptase inhibitors are minimal interactions because these agents are not eliminated by the CYP450 system, nor do they induce or inhibit CYP450 enzymes [26]. The primary metabolic pathway of raltegravir (RAL) is a glucuronoconjugation that primarily involves the uridine diphosphate glucuronosyltransferase (UGT)1A1 enzyme [27]. RAL is neither a substrate nor an inhibitor/inducer of cytochrome CYP450 [28]. Dolutegravir (DTG) is metabolized by UGT1A1 and, to a lesser extent, by CYP3A4 [29]. Clearance of bictegravir (BIC) is primarily hepatic through UGT1A1 glucuronidation and CYP3A4 oxidation [30]. Albuvirtide (ABT) is mainly metabolized through catabolism. DTG and BIC, with a higher drug-resistance barrier than RAL, are the most common second-generation INSTIs available during the study period. In this study, chemotherapy drugs mainly included platinum and fluorouracil drugs, and oxaliplatin undergoes rapid and extensive nonenzymatic biotransformation and is unlikely to be altered by antiviral drugs [31]. 5-FU metabolism is mainly related to dihydropyrimidine dehydrogenase in the liver tissue and is discharged by the kidney. Capecitabine is converted to fluorouracil in tumor tissues via a 3-step enzymatic pathway [32]. Therefore, theoretically, there is a low risk of DDIs between antineoplastic drugs containing fluorouracil and platinum drugs and antiviral drugs. However, we should still pay attention to the fact that a number of antiviral drugs have toxicities that overlap those common to chemotherapy agents, such as myelosuppression, neuropathies, nausea, and diarrhea. In this study, 8 patients (6 patients received Lamivudine/Tenofovir/Efavirenz, and 2 patients received Lamivudine/Tenofovir/Lopinavir/Ritonavir) were excluded because of Inappropriate antiviral regimens. Overall, chemotherapy was still well-tolerated in these 8 patients. Only 1 patient on the regimen of Lamivudine/Tenofovir/Lopinavir/Ritonavir developed severe nausea/vomiting and myelosuppression. Therefore, protease inhibitor-based regimens should be taken with caution in these patients, which is consistent with other findings [33, 34].

Previous experiences support the use of RAL in patients with HIV and concomitant cancer. Our study shows that DTG- or BIC-containing ART regimens are also adequate options in people living with HIV and concomitant CRC in terms of viral logical suppression, lack of additive toxicity, a lower drug-resistance barrier and a lower risk of DDIs. However, in some patients, the CD4 count decreases during chemotherapy, so we should pay attention to the occurrence of opportunistic infections in patients with low baseline CD4 counts. The limitations of our study are primarily related to retrospective observational studies and a relatively small sample size.

## Data Availability

The data that support the findings of this study are available from the corresponding author upon reasonable request.
